# Teaching medical students about dementia: A brief review

**DOI:** 10.1590/1980-57642015DN92000002

**Published:** 2015

**Authors:** Alessandro Ferrari Jacinto, Ananda Ghelfi Raza Leite, José Luiz de Lima Neto, Edison Iglesias de Oliveira Vidal, Paulo José Fortes Villas Bôas

**Affiliations:** 1 Médico, Professor Assistente da Disciplina de Geriatria do Departamento de Clínica Médica da Faculdade de Medicina de Botucatu – Universidade Estadual Paulista Júlio de Mesquita Filho (UNESP).; 2 Psicóloga, Mestranda do Programa de Pós-Graduação em Saúde Coletiva da Faculdade de Medicina de Botucatu – Universidade Estadual Paulista Júlio de Mesquita Filho (UNESP).; 3 Acadêmico do 5º ano da Faculdade de Medicina de Botucatu – Universidade Estadual Paulista Júlio de Mesquita Filho (UNESP).

**Keywords:** dementia, aged, teaching, medical schools, Brazil

## Abstract

Underdeveloped nations have the largest absolute number of the world's elderly population. Approximately 10.7% of the Brazilian population comprises aged individuals. Aging populations are associated with a higher incidence of chronic degenerative diseases such as dementia. Demented individuals place a high burden of care on healthcare systems and family members. General practitioners should be able to diagnose the most common elderly diseases such as dementia since they act as gatekeepers to specialized care. In Brazil, many medical students work as general practitioners upon graduating. The present study shows some scenarios of medical schools worldwide, including Brazilian, regarding teaching on dementia.

## Abstract (pt)

Países subdesenvolvidos têm o maior número absoluto de idosos do mundo. Cerca de 10,7% da população brasileira é constituída por indivíduos com idade igual ou superior a 60 anos. Como o envelhecimento populacional, uma maior incidência de doenças crônico-degenerativas, como a demência, é observada. Indivíduos com demência demandam múltiplos cuidados por parte dos sistemas de saúde e de suas famílias. Os clínicos gerais devem ser capazes de diagnosticar as doenças mais comuns nos idosos, incluindo as demências, já que são eles os profissionais que mais atendem esta população. No Brasil, muitos estudantes de medicina começam a trabalhar como médicos generalistas logo após a graduação em medicina. O presente estudo mostra alguns cenários de escolas médicas do mundo, incluindo algumas brasileiras, acerca do ensino de demências.

## INTRODUCTION

Underdeveloped nations have the largest absolute number of the world's elderly population.^1^ Approximately 10.7% of the Brazilian population comprises aged individuals.^2^

Aging populations are associated with a higher incidence of chronic degenerative diseases such as dementia. Dementia due to Alzheimer's disease is the most common cause of dementia.^3^ An estimated 35.6 million people are living with dementia worldwide. In Brazil, the figure is believed to be around one million individuals.^4^

Several lines of evidence support the notion that the phenomenon of population aging has not been accompanied by the necessary changes in several sectors of society ranging from urban architecture to the education of healthcare professionals.^1,5,6^

Demented individuals place a high burden of care on health care systems and family members. "Neurologists, geriatricians and psychiatrists are usually recognized as specialists in the management of patients with dementia. However, it is also believed that general practitioners should be able to diagnose the most common forms of dementia since they act as gatekeepers to specialized care and are responsible for most of the encounters between patients and the healthcare system.^7^ Hence, it is important to consider whether Brazilian medical schools have been educating their students sufficiently on the care of dementia patients. This is especially relevant in Brazil because many Brazilian medical students start working in primary care units upon graduating and current governmental policies^8^ are likely to increase this trend.

The aim of this study was to briefly review dementia teaching at medical schools in Brazil and abroad.

## DEMENTIA TEACHING IN MEDICAL SCHOOLS

**Around the world.** In 1999, the World Health Organization and the International Federation of Medical Students' Associations carried out a survey on the geriatrics-related content within the curricula of 161 Medical Schools from 36 countries.^9^ Remarkably, 19% of the schools from high-income countries and 38% in developing countries did not report any training in geriatric medicine. Unfortunately, Brazil was not included in the study. Although Geriatrics is not the only medical specialty involved in the care of demented individuals, the study was especially valuable because it provided insight into the importance given to the care of older adults in several international medical schools in the beginning of the twenty-first century.

Hasselbach et al.^10^ in 2007 showed that dementia teaching, as assessed by the presence of obligatory lectures and/or courses, was present in the curricula of all 25 European countries assessed in their study. Notwithstanding, the amount of time dedicated to teaching about dementia has been decreasing gradually over the years, from medical school to specialization in neurology, being even less frequent during the training of certified neurologists.

In 2005, a survey on dementia education and training was conducted in member countries of the European Alzheimer's Disease Consortium (EADC). The study concluded that, in most countries, much needed to be accomplished in order to attain good standards regarding the education of health care professionals about dementia. According to the study, specific educational programs within medical schools targeting the diagnosis and management of dementia existed only in France and the United Kingdom. Another survey conducted in 2008 examined the amount of time dedicated to learning about dementia in another sample of medical schools from EADC countries. The findings revealed that, of the sample of participating medical schools, all dedicated at least a few hours in their curricula to teaching students about dementia. The mean amount of time dedicated to this task was 12.3 hours and ranged from only three hours in Switzerland to 30 hours in Sweden.^11^

In the United Kingdom, General Practitioners are responsible for the care of the majority of people diagnosed with dementia. In 2009, the UK National Dementia Strategy highlighted several deficiencies concerning the knowledge and skills of primary care professionals regarding the care of patients with dementia.^12^ Also in the UK, several specialty societies have been lobbying for mandatory inclusion of education about dementia in the curricula of all medical schools.^13^ These data are especially relevant, not only because the UK is recognized for its high standards in medical education, but also because it represents one of the most important examples of a public health care system that has been strongly centered around primary care.^14^

Tullo et al.,^15^ after applying a questionnaire about teaching and learning on dementia to 23 of the UK´s 31 medical schools, found that 96% delivered this knowledge through clinical rotations, 87% via lectures, 78% seminars, 74% case studies, 61% home/community visits and 9% through problem-based learning. The same study observed a lack of consensus among medical schools on how to teach dementia.

In the United States, there have been difficulties implementing the teaching of health issues that commonly affect the elderly in the curricula of medical schools. In 2007, the Association of American Medical Colleges and the Hartford Foundation established a minimum number of topics in Geriatrics, including dementia, to be mandatory during doctors' medical training.^16^

**In Brazil.** Akin to most countries worldwide, there is scant information on the education given to medical students about dementia in Brazilian medical schools. One report from a survey applied in a public medical school in the State of Minas Gerais showed that 61.7% of its medical students did not receive any education in Geriatrics.^17^ Another study showed that nationwide, 42% of Brazilian medical schools offered teaching in Geriatrics, whereas in the southeast region, with the largest elderly contingent in Brazil, 36% of institutions taught Geriatrics to their students.^18^

According to the document on which the revalidation process of foreign doctors' medical certificates is based, dementia is a mandatory topic in Geriatrics, Neurology and Neurosurgery. This document has been a reference for curricula reform by Brazilian medical schools.^19^ A recent document on Brazilian medical schools' curricula states that students must learn how to manage diseases prevalent during an individual's lifespan - from newborn to elderly. These two documents confirm the need for good quality teaching on dementia in Brazilian medical schools.^20^

**Final considerations.** Medical schools should focus efforts in order to offer multitasking teaching (lectures, seminars and clinical practice) on dementia to their medical students, since many of them will work as general practitioners upon graduating.

Awareness has been raised on the aging population as well as on granting rights to the elderly, for instance through the creation of the "Elderly Statute" which ensures specialized health care for these individuals. However, are we aware of the fact that trained health professionals must exist in order to put elderly's recently acquired rights into practice?
